# CURE: a phase-based therapeutic framework for chronic inflammation

**DOI:** 10.3389/fimmu.2026.1826568

**Published:** 2026-06-15

**Authors:** Nils Kurzen, Stefan Weißinger, Hjalmar Kurzen

**Affiliations:** 1 Department of Dermatology and Allergy, LMU Hospital, Munich, Germany; 2 Department of Dermatology and Allergy, Klinikum rechts der Isar, Technical University of Munich, Munich, Germany; 3 Haut- und Laserzentrum Freising, Freising, Germany

**Keywords:** classification, hypothesis, inflammation, skin disease activity, therapy algorithm

## Abstract

**Background:**

Chronic inflammation represents a central mechanism underlying the persistence of immune-mediated diseases. Unlike acute inflammation, which resolves through coordinated regulatory checkpoints and active resolution programs, chronic inflammation stabilizes in pathological states maintained by cytokine redundancy, tissue-resident immune memory, and self-reinforcing feedback loops that resist termination.

**Conceptual framework:**

Here, we introduce the CURE framework (CONTROL – UNLOAD and RESET – EQUILIBRATE), a phase-based therapeutic model grounded in systems immunology and nonlinear dynamics. The acronym is used as a mnemonic for the proposed therapeutic sequence and does not imply curative eradication of chronic inflammatory disease. Rather, the framework addresses durable disease control, relapse prevention, and stabilization of functional remission. CURE is proposed as a conceptual, phase-based framework that interprets treatment as dependent on the cumulative intensity and duration of anti-inflammatory therapeutic exposure across three clinically relevant phases. Within this hypothesis, CONTROL refers to sufficiently intensive induction therapy intended to suppress self-sustaining inflammatory circuits and potentially cross clinically relevant inflammatory thresholds. UNLOAD and RESET describe a structured, guided de-escalation phase that consolidates remission while restoring endogenous resolution mechanisms. EQUILIBRATE represents proactive, low-amplitude maintenance aimed at stabilizing long-term immune equilibrium and preventing relapse.

**Systems perspective and evidence synthesis:**

From a complex systems perspective, durable disease control may depend not solely on dose selection but also on whether therapeutic exposure is sufficient to suppress feedback-driven immune networks below clinically relevant thresholds. The CURE framework integrates mechanistic and clinical evidence across multiple immune-mediated conditions—including atopic dermatitis, psoriasis, inflammatory bowel disease, and rheumatoid arthritis—to illustrate shared immunodynamic principles underlying durable disease control.

**Conclusions:**

By aligning therapeutic intensity with the dynamic state of the immune system, CURE may provide a transferable, mechanism-oriented framework for personalized intervention in chronic inflammation. It reframes disease management from reactive symptom driven suppression toward proactive, phase-specific immune stabilization. It further offers a common conceptual language across specialties, supporting functional remission, relapse prevention, and long-term tissue integrity.

## 1 Introduction

Inflammation is a fundamental biological response to infection, injury, or environmental insult. In its acute form, it is tightly regulated and typically self-limited: innate immune cells are recruited, pro-inflammatory mediators clear the trigger, and resolution programs restore balance. This resolution requires termination of neutrophil influx, efferocytosis of apoptotic cells, and a lipid mediator switch toward specialized pro-resolving mediators (SPMs) such as lipoxins, resolvins, protectins, and maresins (1).

When resolution fails, acute inflammation can become chronic. Factors like genetic predisposition, autoimmunity, barrier dysfunction, or persistent microbial exposure may lead to sustained cytokine loops and immune memory beyond the original trigger (2). The result is a self-perpetuating inflammatory state that transforms a protective response into a disease-driving process.

Chronic inflammatory diseases such as atopic dermatitis (AD), psoriasis, hidradenitis suppurativa (HS), inflammatory bowel disease (IBD), and rheumatoid arthritis (RA) often follow a relapsing–remitting course. Despite major progress with targeted biologics and small molecules, durable remission remains difficult when therapy is reactive and symptom-focused. Treatment reduction or discontinuation frequently results in relapse, underscoring the need for a structured, phase-based approach that accounts for threshold dynamics and guides individualized adaptation beyond fixed timepoints or symptom-driven decisions alone (3, 4).

In this paper, we define immunological tipping points as non-linear threshold in the inflammatory system at which incremental increases in effective therapeutic exposure produce a disproportionate and sustained reduction in inflammatory self-amplification. Operationally, this corresponds to a transition from a high-relapse to a low-relapse regime, observable as a marked drop in relapse hazard once objective inflammatory activity falls below a critical level.

### 1.1 Illustrative disease–treatment scenarios within the CURE framework

To make the dynamic logic of the CURE framework more tangible, we describe six illustrative disease–treatment scenarios. These scenarios are not intended as a separate classification system, diagnostic taxonomy, or additional conceptual framework. Rather, they are schematic examples of how CONTROL, UNLOAD and RESET, and EQUILIBRATE may become clinically visible across different inflammatory disease courses and treatment scenarios. Their purpose is to support interpretation of the CURE framework by translating its abstract phase structure into recognizable clinical patterns. Six broad scenarios can be distinguished:

1. Self-limited resolution: Acute inflammation terminates once the trigger is cleared, with endogenous resolution programs restoring homeostasis. Examples include uncomplicated wound healing, transient infection-associated inflammation, or acute urticaria, in which endogenous resolution programs restore tissue homeostasis (1).
2. Resolution Failure leading to chronicity: Resolution mediators are deficient or impaired, and inflammatory loops persist. This pattern has been described across several chronic inflammatory diseases, including rheumatoid arthritis, Crohn’s disease, and asthma, where defective efferocytosis, impaired lipid mediator pathways, or persistent cytokine circuits contribute to chronic inflammation (5, 6).
3. Complete therapeutic success: Decisive induction, structured tapering, and proactive maintenance extinguish the “fire” and restore long-term balance. In psoriasis, patients achieving PASI 100 under IL-23 blockade sustain remission with reduced tissue resident memory T cell (T_RM_) formation (7). In ulcerative colitis (UC), deep mucosal healing predicts durable remission and lower colectomy rates (8).
4. Induction failure: Therapy is too weak or too late to cross the first tipping point, leaving inflammatory circuits intact. In Crohn’s disease, infliximab trough levels below 3–5 mg/L fail to saturate TNF receptors (9). Analogously, in asthma, symptom relief without sufficient early anti-inflammatory suppression may mask persistent airway inflammation and predispose to recurrent exacerbations (10–12).
5. Incomplete resolution: This pattern has been described across several chronic inflammatory diseases, including rheumatoid arthritis, Crohn’s disease, and asthma, where defective efferocytosis, impaired lipid mediator pathways, or persistent cytokine circuits contribute to chronic inflammation (13–15).
6. Maintenance failure: Despite apparent clearance, inadequate long-term therapy allows relapse. In AD, proactive twice-weekly corticosteroids halve relapse risk compared with reactive use (16). In IBD, biologic withdrawal after mucosal healing leads to high relapse rates (17). Similar relapse dynamics are observed in asthma when proactive controller-based maintenance therapy is reduced or discontinued (10).

**Figure 1:**
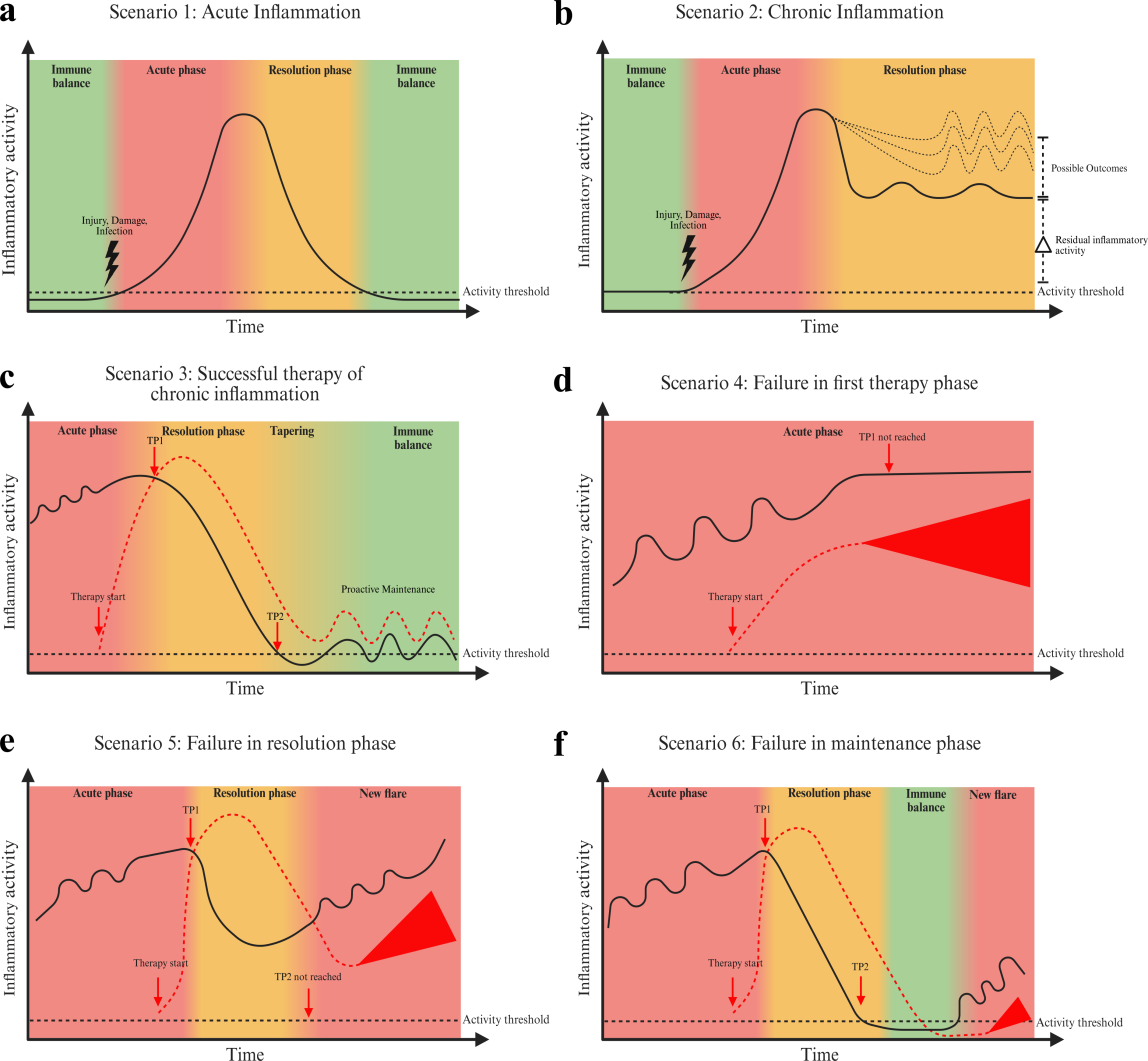
Proposed Archetypes of Inflammation-Treatment Interaction Trajectories. The figure illustrates six simplified inflammation trajectories representing common clinical scenarios across acute and chronic inflammatory diseases. Disease trajectories are shaped by tipping points (TP), defined here as non-linear immunological thresholds at which sufficiently strong or sustained intervention induces a qualitative shift in inflammatory network dynamics rather than incremental symptom improvement. **(a)** Self-limited resolution: acute inflammation resolves spontaneously once the trigger is removed and endogenous resolution programs restore homeostasis. **(b)** Failed resolution leading to chronicity: insufficient counterregulation prevents resolution, allowing self-sustaining inflammatory feedback loops to stabilize and drive persistent disease activity. **(c)** Complete therapeutic success: decisive induction exceeds a critical inflammatory tipping point (TP), collapses self-amplifying immune circuits, and enables transition from active disease to a low-relapse state; subsequent consolidation and maintenance stabilize longterm remission. **(d)** Induction failure: therapeutic intensity or duration is insufficient to cross the initial tipping point, resulting in partial symptom improvement without durable disease control. **(e)** Incomplete resolution: clinical improvement is achieved, but residual subclinical inflammatory activity persists, predisposing to relapse during deescalation. **(f)** Maintenance failure: initial control is achieved, but inadequate long-term stabilization allows reactivation of inflammation and disease recurrence. Together, these trajectories illustrate that durable remission reflects a threshold-dependent state transition rather than linear suppression of inflammation. In panels **(c–f)**, the black solid line represents inflammatory activity, whereas the red dashed line represents effective therapeutic pressure. The figure is schematic and conceptual: treatment intensity must be sufficient to overcome the inflammatory drive and suppress inflammatory activity below the activity threshold. The relevant endpoint is the fall of the black curve below the activity threshold, not a quantitative difference between the red and black curves.

Taken together, these illustrative trajectories show that clinical disease courses are shaped by both intrinsic inflammatory dynamics and the timing, intensity, and structure of anti-inflammatory treatment. They should be understood as explanatory vignettes within CURE, not as an independent taxonomy requiring separate validation. Durable disease control therefore requires not only suppression of the initial inflammatory flare, but also consolidation of remission and prevention of treatment-associated or spontaneous relapse. **Figure 1** summarizes these illustrative disease–treatment scenarios within the CURE framework.

We hypothesize that durable remission reflects a threshold-dependent state transition in inflammatory network dynamics: partial suppression may improve symptoms without changing the underlying attractor, whereas sufficiently deep and sustained suppression can reduce relapse propensity. However, current paradigms such as treat-to-target/tight control and induction–maintenance frameworks predominantly specify what to aim for and how to monitor, without explicitly formalizing (1) phase-transition thresholds, (2) consolidation as a distinct biological objective, and (3) predictable failure modes of de-escalation and maintenance across indications. This leaves step-down decisions and long-term stabilization strategies incompletely standardized across indications.

Here, we introduce the CURE framework (CONTROL – UNLOAD and RESET – EQUILIBRATE) as a phase-structured, threshold-oriented model that links systems-immunology tipping-point dynamics to clinically actionable decision points. The term CURE is used solely as a mnemonic for the proposed therapeutic sequence and should not be interpreted as implying that chronic inflammatory diseases can generally be cured or eradicated. Rather, the framework focuses on durable inflammatory control, relapse-risk reduction, and maintenance of functional remission. Accordingly, CURE should be understood as a hypothesis-generating framework rather than as a validated therapeutic algorithm. The evidence summarized below is used to illustrate the plausibility of phase-dependent inflammatory control and to derive testable predictions, not to prove the model. The model distinguishes two clinically relevant state transitions: Tipping point 1 (TP1), the threshold at which inflammatory activity falls below the level required to sustain clinical disease (clinical control), and tipping point 2 (TP2), a deeper system transition reflecting restoration of regulatory equilibrium (deep remission). The framework further predicts hysteresis between induction and maintenance, such that once TP2 has been reached, stable disease control can be maintained with substantially lower therapeutic exposure.

This yields concrete research implications: phase-specific operational endpoints, biomarker and pharmacokinetic/pharmacodynamic (PK/PD) proxies for TP2, and falsifiable predictions for trial design and relapse-risk stratification, which are summarized in **Box 1**.

Box 1 **Consolidated testable predictions and falsifiability criteria derived from CURE**

**Prediction 1: TP1 — threshold-like exposure–response for clinical control**

During CONTROL, crossing TP1 is predicted to occur when therapeutic exposure reaches a minimum effective level sufficient to suppress dominant inflammatory amplification loops. Above this threshold, objective disease activity and symptom burden should decline disproportionately compared with incremental increases in exposure. Here, pathway-suppression/target-engagement metrics refer to disease-appropriate objective readouts of inflammatory suppression or pharmacodynamic target engagement, such as trough concentrations, receptor occupancy, time above a pharmacodynamic threshold, biomarkers such as CRP, fecal calprotectin, FeNO, or validated disease activity scores where direct biological markers are unavailable.

Test: Model nonlinear or change-point relationships between these metrics and rapid clinical control after induction.

**Prediction 2: Early depth of response during CONTROL predicts later stability**

Earlier and more complete CONTROL is predicted to reduce subsequent relapse hazard beyond what is explained by short-term symptom improvement alone. Here, depth of response refers to the magnitude and completeness of early disease suppression at a predefined induction timepoint, assessed using stringent disease-specific targets. Examples include PASI100 or PASI90 in psoriasis, EASI90 or EASI75 in atopic dermatitis, mucosal healing plus biomarker normalization in IBD, or DAS28 remission with low acute-phase reactants in RA.

Test: Compare relapse hazard and maintenance requirements according to predefined depth-of-response categories and time-to-control, adjusting for baseline severity and adherence.

**Prediction 3: Deep remission is a prerequisite for successful de-escalation**

De-escalation undertaken after higher levels of deep remission is predicted to have lower relapse rates than de-escalation after clinical control alone. Here, levels of deep remission refer to the degree of residual inflammatory quiescence at taper start, assessed by disease-specific combinations of clinical control, biomarker normalization, tissue healing, imaging, or functional measures.

Test: Conduct tapering studies stratified by levels of deep remission at de-escalation start and compare subsequent relapse rates.

**Prediction 4: Residual activity identifies clinical, but not deep remission**

Patients with residual inflammatory activity despite clinical improvement represent clinical, but not deep remission, and are predicted to relapse more frequently unless UNLOAD/RESET is completed.

Test: prospective monitoring of residual markers during taper; association with subsequent flare-days/relapse.

**Prediction 5: Proactive maintenance reduces cumulative disease burden**

EQUILIBRATE strategies using proactive maintenance and structured monitoring will reduce flare-days, rescue medication use, and cumulative inflammatory burden compared with reactive retreatment.

Test: pragmatic trials comparing proactive maintenance protocols vs symptom-triggered strategies.

**Prediction 6: Cross-indication convergence of phase dynamics**

Across different chronic inflammatory diseases, the same phase dynamics will be observable: threshold-crossing induction, consolidation of deep remission, and stabilization under minimal-effective maintenance.

Test: harmonized endpoints across 2–3 indications; compare phase-transition success rates and relapse hazard.

**Prediction 7: Failure modes are phase-specific and predictable**

Relapse will cluster into identifiable failure modes: insufficient CONTROL (no threshold crossing), incomplete RESET, corresponding to clinical, but not deep remission, or inadequate EQUILIBRATE (maintenance failure).

Test: classify patients by phase-specific endpoint failure and validate differential relapse patterns and re-induction responsiveness.

**Falsifiability criteria**

The CURE hypothesis would be disproven if (1) no threshold-like relationships can be demonstrated between TP1-directed pathway-suppression/target-engagement metrics and clinical control after induction, (2) deep-remission markers do not stratify relapse risk or de-escalation success, and (3) proactive maintenance does not reduce cumulative flare burden relative to reactive care when tested prospectively across more than one indication.

## 2 The CURE framework – a three-phase conceptual model for inflammation control

Clinical observations and systems-immunology concepts suggest that chronic inflammation can behave as a nonlinear dynamic system characterized by feedback amplification, redundancy, and immune memory. In such systems, small perturbations may have limited effects, whereas sufficiently strong or sustained interventions may shift disease activity into a more stable low-inflammatory state (18). Within this hypothesis, durable disease control may depend not only on suppressing symptoms but also on reducing inflammatory activity below clinically relevant thresholds (19).

Mathematical modeling provides a mechanistic basis for this behavior. In the reduced model of the acute inflammatory response developed by Reynolds et al., three interacting variables—pathogenic stimulus, pro-inflammatory mediators, and counter-regulatory anti-inflammatory factors—generate multiple stable steady states (19). Depending on feedback strength and timing, the system may settle into health, sustained inflammation, or immune paralysis. The model predicts that minor modulation near equilibrium often fails to alter disease trajectory, whereas decisive, well-timed intervention can push the system across a bifurcation threshold back toward homeostasis. Conversely, premature withdrawal or excessive suppression may drive the system into an immunosuppressed “cold” attractor. These nonlinear dynamics offer a mechanistic explanation for delayed therapeutic responses, apparent resistance, and rebound phenomena following dose reduction.

In this context, we use the term *therapeutic exposure* to describe the cumulative intensity, timing, dosing frequency, and duration of treatment required to overcome dominant inflammatory feedback loops. Operationally, this includes cumulative exposure (area under the concentration–time curve), peak concentration, receptor occupancy, dosing frequency, and duration of intervention—parameters that together determine whether a biological threshold is crossed (9, 20, 21).

The CURE model (CONTROL–UNLOAD and RESET–EQUILIBRATE) seeks to translate threshold biology into a practical, phase-based therapeutic framework. Conceptually transferable across chronic inflammatory diseases, CURE reframes therapy as an exposure- and timing-dependent sequence: a decisive induction phase to cross the inflammatory tipping point, a structured de-escalation phase to consolidate remission, and a proactive maintenance phase to stabilize long-term immune balance (16).

### 2.1 Phase 1: CONTROL

Conceptually, CONTROL refers to the induction phase in which therapeutic intensity is sufficient to suppress dominant inflammatory amplification loops. In relapse-prone immune-mediated diseases, partial inhibition may improve symptoms while leaving residual cytokine activity, tissue inflammation, or immune memory capable of reigniting disease. Within the CURE hypothesis, effective CONTROL therefore requires not only symptomatic improvement but a depth and duration of suppression sufficient to shift the system from a high-inflammatory state toward a controlled, lower-relapse state.

This phase is grounded in the concept that chronic inflammation can be maintained by positive feedback between cytokine release, barrier disruption, immune-cell recruitment, and tissue-resident inflammatory memory. Pattern-recognition pathways activated by pathogen- or damage-associated signals, as well as cytokine networks involving TNF-α, IL-1, IL-6, IL-17, IL-23, or type 2 cytokines, may contribute to such self-amplifying loops depending on the disease context. However, these pathways should be understood as illustrative examples rather than as evidence for a single universal mechanism across all chronic inflammatory diseases (22–28).

Several clinical and translational observations are consistent with this threshold-oriented view. Exposure–response relationships for anti-TNF therapy in Crohn’s disease suggest that insufficient trough levels are associated with loss of response, whereas higher target exposure correlates with more durable remission (9). In psoriasis, more complete blockade of synergistic inflammatory pathways, such as dual IL-17A/F inhibition, is associated with higher complete-clearance rates than selective IL-17A inhibition alone (29). Early-intervention data with IL-23 blockade further suggest that deeper initial suppression may reduce relapse-associated tissue-resident memory signatures (7, 13, 14). These examples support the plausibility of the CONTROL concept, but they do not prove a universal tipping-point mechanism.

Thus, CONTROL is best understood as a proposed front-loaded induction phase intended to suppress self-perpetuating inflammatory circuits, reduce residual inflammatory activity, and create the conditions for subsequent consolidation in the UNLOAD and RESET phase.

### 2.2 Phase 2: UNLOAD and RESET

Once acute inflammation is effectively suppressed, the second phase of CURE focuses on gradual therapeutic de-escalation. The goal is to reduce treatment intensity without risking relapse, maintaining immunological balance while tapering active intervention. This is not a passive decline but an actively guided consolidation phase: therapy is lowered in a structured fashion with pre-defined intervals, objective monitoring, and readiness to re-escalate if necessary (15). Clinically, this stepwise reduction may include lowering corticosteroid potency or frequency, extending dosing intervals of biologics, or switching to non-steroidal maintenance such as topical calcineurin inhibitors in AD (30).

A central distinction in this phase is the difference between clinical remission and deep remission. Clinical remission refers to symptom-based or score-based disease control, which may still coexist with residual biological activity. By contrast, deep remission is defined here as sustained clinical control together with objective evidence of low inflammatory activity, such as normalization of validated biomarkers and/or tissue-, imaging-, or function-based measures appropriate to the disease context. Within the CURE hypothesis, de-escalation during clinical but not biological remission is predicted to carry a higher relapse risk, because residual cytokine activity, tissue-resident immune memory, or unresolved barrier damage may persist below the threshold of clinical detection (13, 15, 31–34).

From an immunobiological perspective, UNLOAD and RESET represents a proposed consolidation window after initial inflammatory suppression. Pathogenic networks may have been reduced, but endogenous resolution programs and tissue integrity may not yet be fully restored. Importantly, resolution of inflammation is not merely passive decay; it involves active processes such as cessation of neutrophil influx, efferocytosis of apoptotic cells, macrophage phenotype switching, and a lipid mediator shift toward specialized pro-resolving mediators including lipoxins, resolvins, protectins, and maresins (1). Defects in resolution pathways, including impaired lipoxin or annexin A1 signaling and defective efferocytosis, have been implicated in chronic inflammatory disease and provide a mechanistic rationale for treating consolidation as a distinct biological objective (2, 35, 36).

Clinical examples consistent with this concept include biomarker-guided de-escalation in Crohn’s disease, criteria-driven tapering in rheumatoid arthritis, and proactive step-down topical strategies in atopic dermatitis (15, 30, 37, 38). Across these contexts, de-escalation appears safest when clinical control is accompanied by objective evidence of low residual inflammatory activity. Conversely, abrupt withdrawal or tapering before adequate consolidation is expected to increase relapse risk, although the precise markers defining safe de-escalation remain disease-specific and incompletely validated. Importantly, routinely used clinical activity instruments should not be equated with deep-remission markers. Scores such as EASI, SCORAD, PASI, or DAS28 are valuable for standardized assessment of clinical disease activity and for detecting early clinical worsening during tapering, but they do not necessarily capture residual subclinical inflammatory activity. By contrast, deep-remission markers should ideally reflect biological quiescence more directly, for example through validated biomarker normalization, imaging-, endoscopic-, histological-, functional-, or tissue-based measures, depending on the disease context. For many indications, especially in dermatology, such markers remain incompletely developed. Future instruments will need to be sensitive to subclinical inflammation, reproducible across time and centers, responsive during de-escalation, feasible for routine monitoring, and predictive of relapse risk after dose reduction or interval extension.

Juvenile idiopathic arthritis provides a pediatric example of this principle. Treat-to-target recommendations in JIA define remission as the primary therapeutic target, and the PREVENT-JIA trial showed that biomarker-guided withdrawal using S100A12 and high-sensitivity CRP may reduce flare risk in children with JIA in clinical remission. This supports the concept that safe de-escalation may require evidence of suppressed residual inflammatory activity beyond clinical remission alone (39, 40).

The proposed operational endpoint of Phase 2 is deep remission: sustained clinical control plus at least one disease-appropriate objective marker or proxy of low residual inflammatory activity, while recognizing that such markers are not equally validated across indications. By consolidating the gains of CONTROL while allowing partial restoration of resolution pathways and tissue stability, UNLOAD and RESET is proposed to create the conditions for successful EQUILIBRATE. Poorly timed or insufficiently monitored tapering, by contrast, may undo the benefits of induction and increase subsequent flare burden.

### 2.3 Phase 3: EQUILIBRATE

Even after clinical remission has been achieved, many chronic inflammatory diseases remain relapse-prone. Residual tissue-resident immune cells, autoreactive B-cell clones, persistent barrier dysfunction, microbial or environmental triggers, and subclinical inflammatory activity may continue to lower the threshold for disease reactivation (41–43). Within the CURE framework, EQUILIBRATE refers to the proposed maintenance phase in which the objective is not curative eradication, but stabilization of a state of low risk of relapse under the minimum effective long-term treatment burden.

EQUILIBRATE is conceptualized as a proactive, low-amplitude maintenance strategy intended to prevent re-entry into a self-amplifying inflammatory state. This may involve continued low-dose topical therapy, scheduled biologic or conventional systemic maintenance, controller-based anti-inflammatory treatment, or structured biomarker and symptom surveillance, depending on the disease context. The central principle is that treatment intensity after remission should be sufficient to preserve stability, but not higher than necessary to maintain functional remission.

Mechanistically, EQUILIBRATE reflects a dynamic balance between three interacting variables: residual inflammatory activity, recurrent environmental or microbial trigger pressure, and the intensity of the maintenance intervention. Environmental and disease-specific triggers such as allergens, mechanical irritation, microbial dysbiosis, infections, barrier disruption, or tissue stress may act as recurrent perturbations that push the system back toward inflammatory activation. Whether these perturbations result in overt relapse depends partly on the remaining inflammatory substrate, including tissue-resident immune memory, autoreactive cell populations, unresolved barrier damage, or low-grade cytokine activity. Maintenance therapy can therefore be understood as a stabilizing buffer that counteracts both residual inflammatory activity and external trigger pressure. If trigger burden or subclinical inflammation remains high, a stronger or more continuous maintenance signal may be required; if tissue integrity is restored and residual activity remains low, lower-intensity maintenance or cautious interval extension may be feasible. These relationships are likely disease- and patient-specific and are unlikely to be captured by a single routine-care measure. However, several components may be monitored longitudinally. Candidate pre-emptive markers would be those that rise before overt clinical relapse and thereby indicate early reactivation of the inflammatory system. Examples include fecal calprotectin and CRP in inflammatory bowel disease, FeNO or eosinophil-associated measures in asthma, S100A12 and high-sensitivity CRP in juvenile idiopathic arthritis, and ultrasound-detected subclinical synovitis in rheumatoid arthritis (39, 44, 45). In dermatological diseases, comparable relapse-predictive markers are less established, but minimally invasive approaches such as tape-strip transcriptomic or proteomic profiling may help detect residual cutaneous inflammation or barrier dysfunction in future studies (46).

A clinically useful EQUILIBRATE instrument would need to be reproducible, feasible for repeated use, sensitive to subclinical inflammatory reactivation, and predictive of subsequent relapse risk. Accordingly, pre-emptive step-up therapy would refer to a temporary increase in maintenance intensity when validated reactivation markers rise or deviate from the patient’s individual baseline before full clinical relapse occurs. This concept is partly supported in selected indications, but remains exploratory in many inflammatory skin diseases and requires prospective validation. Therefore, EQUILIBRATE should be interpreted as a conceptual stabilization strategy rather than as a uniform therapeutic protocol.

Clinical observations across atopic dermatitis, psoriasis, inflammatory bowel disease, rheumatoid arthritis, and asthma are consistent with the principle that proactive maintenance or structured monitoring can reduce relapse risk compared with purely reactive retreatment (16, 17, 42–51). The specific maintenance strategy differs by disease, ranging from proactive topical therapy in atopic dermatitis to scheduled biologic therapy in psoriasis and inflammatory bowel disease, disease-modifying maintenance therapy in rheumatoid arthritis, or inhaled corticosteroid-containing controller therapy in asthma. These examples support the plausibility of EQUILIBRATE, but they do not establish a universal maintenance threshold across all chronic inflammatory diseases.

The proposed endpoint of EQUILIBRATE is functional remission: sustained low relapse risk, preserved tissue integrity, stable patient-reported outcomes, and acceptable long-term treatment burden. In this sense, EQUILIBRATE represents a shift from episodic, flare-driven treatment toward proactive stabilization of remission, in which maintenance intensity is adjusted to relapse risk, residual inflammatory burden, and trigger exposure, while recognizing that the evidence base and feasible strategies differ substantially between diseases.

### 2.4 Pharmacokinetic and clinical observations consistent with tipping points

The CURE framework uses pharmacokinetic, pharmacodynamic, and clinical observations to formulate a threshold-oriented hypothesis for chronic inflammation. These observations do not prove the existence of universal immunological tipping points. Rather, they suggest that in selected immune-mediated diseases, durable disease control may depend on achieving sufficient pathway suppression for a sufficient duration, rather than on incremental symptom reduction alone.

Pharmacokinetic and pharmacodynamic data provide one line of evidence consistent with this hypothesis. The relevant issue is not therapeutic class per se, but whether a given intervention achieves sufficient depth and duration of pathway suppression within a feedback-amplified inflammatory network. For biologic therapies, target occupancy, tissue exposure, pathway redundancy, and compensatory signaling may all influence whether clinical improvement translates into durable inflammatory control. Physiologically based PK and mechanistic PK/PD models suggest that drug concentrations above *in vitro* potency estimates may be required *in vivo* to maintain sufficient target engagement (21). Clinically, anti-TNF exposure–response data in Crohn’s disease show that infliximab trough concentrations in a defined therapeutic range are associated with better disease control and lower relapse risk than subtherapeutic exposure (9). Similarly, in psoriasis, dual IL-17A/F blockade with bimekizumab achieved higher rates of complete skin clearance than selective IL-17A inhibition, consistent with the concept that more complete suppression of synergistic inflammatory pathways may produce deeper clinical responses (29). These examples are used as illustrative PK/PD observations and do not imply that biologics and small molecules operate in categorically different inflammatory systems.

These examples are compatible with threshold-like exposure–response behavior, but they should not be interpreted as definitive proof of discrete biological phase transitions. The relevant thresholds are likely disease-specific, pathway-specific, and dependent on timing, tissue compartment, disease duration, and residual inflammatory memory. In some diseases, such thresholds may be measurable through pharmacokinetic parameters, biomarkers, imaging, or tissue signatures; in others, they remain theoretical constructs requiring prospective validation.

Analogies from physics and ecology, including phase transitions and ecosystem regime shifts, are useful for illustrating how nonlinear systems can shift abruptly once critical parameters are exceeded (18, 20). However, these analogies are conceptual and should not be used as evidence that chronic inflammatory diseases follow identical transition rules. Within CURE, their role is to provide a systems-level language for generating testable hypotheses about induction depth, consolidation, maintenance, and relapse risk.

Taken together, pharmacokinetic, pharmacodynamic, and clinical observations support the plausibility of threshold-like dynamics in chronic inflammation. They provide a rationale for testing whether early intensive induction, structured de-escalation after objective remission, and proactive maintenance reduce relapse hazard and cumulative inflammatory burden compared with predominantly reactive treatment strategies. Prospective studies using predefined exposure, biomarker, and relapse-risk endpoints are required to determine whether the proposed CURE transitions can be empirically validated.

### 2.5 Operational endpoints, decision points, and testable predictions

A central requirement for a hypothesis-and-theory framework is that it can be operationalized and empirically challenged. The CURE model therefore proposes phase-specific objectives, objective endpoints indicating progression to the next phase, and decision points for escalation, de-escalation, and maintenance adjustment. While the specific markers will differ by disease and tissue compartment, the overarching logic is conserved: CONTROL aims to cross an initial threshold (state transition from active flare to controlled disease), UNLOAD/RESET aims to consolidate deep remission (reducing relapse substrates and residual activity), and EQUILIBRATE aims to stabilize the resolved regime and minimize relapse hazard under a minimal-effective maintenance burden.

To translate this operational logic into a structured research framework, **Table 1** summarizes the three CURE phases according to their primary objective, candidate clinical endpoints, optional mechanistic research endpoints, and illustrative decision rules. The table is not intended as a disease-specific treatment algorithm, but as a generic framework that can be adapted to indication-specific instruments, biomarkers, imaging modalities, tissue readouts, and PK/PD parameters. Importantly, routine clinical activity scores may support monitoring and detection of worsening, whereas decisions about deep remission and safe de-escalation should, where possible, be anchored in objective markers or validated proxies of low residual inflammatory activity.

**Table 1:** Operational framework of the CURE model: phase-specific objectives, candidate endpoints, and illustrative decision rules.

CURE phase	Primary objective	Candidate operational endpoints (core)	Optional mechanistic endpoints (research)	Decision rules (illustrative)
CONTROL	Cross the first threshold: suppress amplification loops and achieve rapid clinical control	• Time-to-control (time to predefined target) • Early trajectory (e.g., % improvement by week 2–4) • Rescue medication avoidance • Feasible inflammation proxies (e.g., CRP where relevant; disease-specific markers)	• Tissue inflammatory signatures • Pathway suppression markers • Target engagement proxies (PK/PD: trough/occupancy)	• Escalate if predefined control targets are not reached within a prespecified time window. • Maintain induction intensity until target attained and early stabilization criteria met.
UNLOAD and RESET	Consolidate deep remission while reducing treatment burden; minimize residual activity and relapse substrates	• Sustained target attainment over a defined consolidation period • Deep remission marker(s) appropriate to disease (e.g., biomarker normalization; functional/tissue healing endpoint) • Absence of clinically relevant flares during taper as a necessary, but not sufficient, safety criterion	• Histologic healing • Tissue-resident memory signatures • Transcriptomics/proteomics substudies	• De-escalate stepwise only if sustained control plus deep remission marker(s) present. • Pause or reverse taper if early reactivation signals emerge.
EQUILIBRATE	Stabilize resolved regime: prevent re-entry into inflammatory state under minimal-effective maintenance	• Sustained absence of clinically relevant flares over a predefined maintenance interval • Stable patient-reported outcomes and tissue/function measures • Longitudinal surveillance markers, including disease-appropriate biomarkers or functional measures • Flares, flare-days, or rescue medication use as *post-hoc* indicators of insufficient maintenance intensity	• Longitudinal immune memory readouts • Microbiome/driver profiling • Digital biomarkers	• Adjust maintenance intensity proactively based on surveillance. • Pre-emptive step-up when validated reactivation markers rise or deviate from the patient’s individual baseline before overt clinical relapse. • Consider de-escalation only after prolonged deep remission with consistently low relapse propensity.

Implementation note: Disease-specific mappings should distinguish routine clinical activity instruments from objective deep-remission proxies. EASI/SCORAD in atopic dermatitis, PASI in psoriasis, DAS28 in rheumatoid arthritis, exacerbation frequency in asthma, and similar scores are useful for standardized monitoring and early relapse detection, but should not be considered sufficient evidence of biological deep remission on their own. Where available, deep-remission assessment should incorporate disease-appropriate objective measures such as biomarker normalization, fecal calprotectin/CRP in IBD, endoscopic or imaging-based healing, histological or tissue-based measures, lung function/FeNO in asthma, or PK/PD and target-engagement proxies. The CURE framework does not prescribe a single instrument; it prescribes a phase logic and highlights the need for validated, relapse-predictive deep-remission markers before meaningful de-escalation. Absence of clinically relevant flares during taper or maintenance should be interpreted as a necessary, but not sufficient, criterion for successful UNLOAD/RESET or EQUILIBRATE. Conversely, flares, increasing flare-days, or rising rescue medication use provide *post-hoc* evidence of insufficient consolidation or maintenance intensity.

CRP, C-reactive protein; DAS28, Disease Activity Score 28; EASI, Eczema Area and Severity Index; FeNO, fractional exhaled nitric oxide; IBD, inflammatory bowel disease; PASI, Psoriasis Area and Severity Index; PK/PD, pharmacokinetic/pharmacodynamic; SCORAD, SCORing Atopic Dermatitis.

Operationalization also clarifies two distinct transitions. TP1 denotes the shift from uncontrolled, self-amplifying inflammation to clinical control—operationally, the point at which a minimum effective exposure drives the system into a controlled regime, yielding a rapid, disproportionate reduction in inflammatory load and symptoms and enabling entry into UNLOAD/RESET. TP2, in contrast, is not defined by symptoms alone: it denotes a deeper transition toward a state of low risk of relapse in which residual inflammatory activity, relapse substrates, tissue instability, and trigger susceptibility are sufficiently reduced to permit durable stabilization. Relapse risk after TP2 is therefore determined not only by treatment intensity, but also by environmental or microbial trigger burden, host-intrinsic susceptibility, tissue integrity, and residual immune memory. Patients may appear clinically clear while residual tissue inflammation, pathogenic memory, barrier disruption, or structural drivers persist, leaving them vulnerable to relapse when treatment is reduced or when trigger pressure increases.

In practice, tipping points can be tested using change-point and nonlinear models that relate objective disease activity, treatment exposure, and trigger-related variables to subsequent relapse risk. For TP1, the prediction is a steep early drop in inflammatory activity and symptom burden once a minimum effective exposure is exceeded. For TP2, the prediction is a distinct inflection: below a critical level of residual inflammatory activity and relapse susceptibility, the hazard of relapse over a predefined follow-up window falls disproportionately. This state of low risk of relapse should remain more stable not only during cautious dose reduction or interval extension, but also under ordinary environmental, microbial, or barrier-related perturbations. Evidence for hysteresis—higher exposure needed to reach TP2 than to maintain stability thereafter—would further support a state-transition mechanism. These signatures can be evaluated prospectively with pre-specified thresholds and validated by prediction of relapse during de-escalation, trigger exposure, or longitudinal maintenance monitoring.

To facilitate testing across indications, we propose a generic endpoint set (**Box 1**) that can be mapped onto disease-specific instruments, biomarkers, imaging, and functional readouts.

### 2.6 Conceptual positioning of CURE in relation to established therapeutic paradigms

CURE is not proposed as an alternative to established therapeutic paradigms such as treat-to-target, tight control, induction–maintenance strategies, de-escalation frameworks, window-of-opportunity concepts, or proactive maintenance therapy. These approaches address different dimensions of inflammatory disease management: treat-to-target and tight-control strategies define therapeutic goals, monitoring intervals, and escalation rules; window-of-opportunity concepts emphasize early intervention; induction–maintenance and de-escalation frameworks structure treatment intensity over time; and proactive maintenance strategies aim to prevent relapse after disease control has been achieved.

By contrast, CURE is a hypothesis-generating conceptual model of how inflammatory activity and therapeutic exposure may interact over time. It does not prescribe specific drugs, biomarkers, or treatment algorithms, and it does not replace disease-specific guidelines. Rather, it provides a phase-based and threshold-oriented language for interpreting why existing strategies may be effective in certain contexts: early intensive treatment may correspond to CONTROL, structured tapering after objective remission to UNLOAD and RESET, and proactive relapse prevention to EQUILIBRATE.

CURE and established therapeutic paradigms should therefore be viewed as complementary. Established paradigms provide clinically actionable strategies, whereas CURE proposes a systems-level interpretation of their timing, sequencing, and potential failure modes. However, CURE remains unvalidated as a universal model and generates testable predictions about relapse risk, de-escalation success, and maintenance requirements rather than defining universally applicable thresholds. **Table 2** summarizes how established therapeutic concepts may map onto specific aspects of the CURE framework.

**Table 2:** Relationship between CURE and established therapeutic concepts.

Concept	Definition/focus	Control	Unload and reset	Equilibrate	Main scope limitation	References
**Treat-to-Target (T2T)**	Definition of explicit treatment targets (e.g. remission or low disease activity) with regular assessment and therapy adjustment until the target is reached.	+	+	–	Defines targets and escalation rules, but provides less guidance on consolidation, tapering, and long-term maintenance after target attainment.	(39, 47, 89)
**Tight control/biomarker-guided management**	Frequent objective monitoring using biomarkers, imaging, or endoscopy, with predefined escalation rules independent of symptoms.	(+)	(+)	(+)	Optimizes monitoring and escalation, but does not itself define phase-specific induction, tapering, or maintenance strategies.	(15, 47)
**Treat-through vs withdrawal**	Explicit distinction between induction therapy and maintenance therapy, with debate over continuation versus withdrawal after response.	(+)	(+)	(+)	Addresses continuation versus stopping therapy, but often provides limited criteria for identifying patients suitable for safe de-escalation.	(48, 49)
**Stepup/Stepdown**	Gradual escalation of therapy in response to disease activity, followed by dose reduction once control is achieved	+	+	–	Structures treatment intensity, but thresholds for safe step-down and relapse prevention are often disease-specific and empirical.	(37, 38)
**“Hit hard and early” strategy**	Early use of high-intensity therapy to rapidly suppress inflammation and prevent disease progression.	+	–	–	Primarily induction-focused; provides limited guidance on post-remission consolidation and maintenance.	(90, 91)
**De-escalation/tapering frameworks**	Structured reduction of therapy intensity to minimize toxicity once disease control is achieved, often guided by biomarkers or imaging.	–	++	(+)	Focuses on reducing treatment burden after control, but does not address how initial control should be achieved.	(37, 38, 40)
**Window of opportunity**	Early aggressive treatment during a presumed critical time window to alter long-term disease trajectory and prevent irreversible damage.	++	(+)	(+)	Emphasizes early intervention, but offers limited guidance for established disease or long-term maintenance.	(90, 91)
**Proactive therapy**	Ongoing low-intensity therapy after disease control to prevent relapse and stabilize remission.	–	(+)	+	Focuses on relapse prevention after control, but does not define induction or structured de-escalation.	(92, 93)

Rating scale: ++ = central focus of the concept; + = explicitly addressed but not the central focus; (+) = indirectly or partially addressed; − = not addressed or not part of the concept’s intended scope.

## 3 Selected clinical applications and boundaries of the CURE framework

The following examples are intended to illustrate how the proposed CURE logic may map onto different inflammatory diseases, not to provide comprehensive disease-specific treatment algorithms. To avoid repetition of the phase definitions presented above, this section focuses on the degree to which CONTROL, UNLOAD and RESET, and EQUILIBRATE are already reflected in existing clinical practice and where important gaps remain. The examples differ substantially in their evidentiary basis: in some diseases, all three phases are supported by treat-to-target strategies, tapering studies, or maintenance data; in others, CURE primarily serves as a conceptual lens to identify unmet needs. A structured comparison is provided in **Table 3**.

**Table 3:** Hypothetical mapping of classical treatment pathways to the CURE framework across chronic inflammatory conditions.

Disease	Classical therapy pathway	Hypothetical CURE-based interpretation (phase-specific)	Reference
Atopic dermatitis	Reactive topical corticosteroids during flares; intermittent TCI in remission.	C: Trigger identification and intensive induction with potent TCS/TCI. U/R: Step-down guided by EASI/POEM. E: Proactive twice-weekly low-dose maintenance.	(62, 93)
Psoriasis	Stepwise escalation (topical → systemic → biologic), discontinuation after improvement.	C: Early high-efficacy biologic induction targeting PASI90/100. U/R: Structured tapering or interval extension in selected responders. E: Sustained maintenance at lowest effective dose/interval.	(62)
Hidradenitis suppurativa	Prolonged escalation from antibiotics to surgery and biologics.	C: Early initiation of biologics to suppress inflammation. U/R: Limited tapering guided by disease activity. E: Long-term maintenance with biologics ± surgery/laser.	(94)
Rheumatoid arthritis	DMARD induction (e.g., methotrexate), possible withdrawal in remission.	C: Rapid remission with combination DMARDs or biologics. U/R: Biomarker- and imaging-guided tapering. E: Maintenance of low disease activity with minimal effective therapy.	(37)
Juvenile idiopathic arthritis	Treat-to-target therapy with DMARDs or biologics; withdrawal in selected patients with remission.	C: Target-driven suppression toward inactive disease. U/R: Biomarker- and imaging-informed withdrawal using S100A12/hsCRP and subclinical synovitis assessment. E: Structured follow-up to preserve remission and detect flare.	(39, 40, 95)
Inflammatory bowel disease	Steroid induction followed by immunomodulators; relapse-driven escalation.	C: Induction of deep remission and mucosal healing. U/R: De-escalation guided by biomarkers and endoscopy. E: Proactive biomarker-driven maintenance with biologics or 5-ASA.	(47)
Bronchial asthma	Reliever-based therapy with stepwise escalation during exacerbations.	C: Early suppression with high-dose ICS or systemic steroids. U/R: Tapering to lowest effective dose guided by ACT. E: Continuous controller-based maintenance therapy.	(96)
Systemic autoimmune/hyperinflammatory conditions	Reactive high-dose steroids and immunosuppressants.	C: Rapid disease control with immunosuppression or biologics. U/R: Careful steroid tapering guided by biomarkers. E: Proactive maintenance to prevent organ damage.	(51)

The CURE-based interpretations shown here are conceptual and hypothesis-generating. They should not be interpreted as validated CURE-guided treatment algorithms or proven advantages of the framework.

ACT, Asthma Control Test; C, CONTROL; DMARD, disease-modifying antirheumatic drug; E, EQUILIBRATE; EASI, Eczema Area and Severity Index; hsCRP, high-sensitivity C-reactive protein; IBD, inflammatory bowel disease; ICS, inhaled corticosteroid; JIA, juvenile idiopathic arthritis; PASI, Psoriasis Area and Severity Index; POEM, Patient-Oriented Eczema Measure; S100A12, S100 calcium-binding protein A12; TCI, topical calcineurin inhibitor; TCS, topical corticosteroid; U/R, UNLOAD and RESET; 5-ASA, 5-aminosalicylic acid.

### 3.1 Diseases with substantial phase alignment

Crohn’s disease and rheumatoid arthritis provide some of the clearest clinical examples consistent with the CURE framework. In Crohn’s disease, early biomarker-guided escalation, objective targets such as mucosal healing, therapeutic drug monitoring, and high relapse rates after biologic withdrawal collectively align with the sequence of induction, consolidation, and maintenance proposed by CURE. The CALM trial showed that biomarker-guided escalation achieved higher rates of endoscopic and clinical remission than symptom-driven management, while STRIDE-II emphasizes objective targets beyond symptom control (15, 47). Therapeutic drug monitoring studies further support the concept that insufficient anti-TNF exposure is associated with loss of response, whereas maintaining defined therapeutic trough concentrations may improve disease control (9). Conversely, withdrawal studies such as STORI and SPARE demonstrate that stopping biologic therapy after apparent remission is associated with substantial relapse risk, supporting the need for proactive maintenance in many patients (48, 49).

Rheumatoid arthritis similarly illustrates the importance of early and sufficiently intensive treatment followed by careful, criteria-driven tapering in selected patients. Early combination therapy trials such as FIN-RACo and SWEFOT support the principle that rapid suppression of inflammation improves long-term outcomes and limits structural damage (50, 51). Once sustained remission is achieved, trials such as HONOR, PRESERVE, and ACT-RAY suggest that dose reduction or discontinuation may be feasible in selected patients, but only under strict remission criteria and close monitoring (52–55). Thus, Crohn’s disease and rheumatoid arthritis do not prove the CURE model, but they provide clinically mature examples in which induction, consolidation, and maintenance can be operationalized using objective endpoints.

### 3.2 Dermatological examples

Dermatological diseases illustrate both the strengths and the current limitations of the framework. In atopic dermatitis, the CURE sequence is most clearly reflected in topical therapy: flares are treated with sufficiently intensive anti-inflammatory induction, followed by step-down therapy and proactive intermittent maintenance. Proactive topical corticosteroid or tacrolimus regimens reduce relapse risk and prolong flare-free intervals compared with purely reactive treatment, supporting the EQUILIBRATE concept in local therapy (16, 56–59). In systemic atopic dermatitis therapy, however, structured de-escalation and biomarker-guided maintenance are less well established. The SOLO-CONTINUE trial showed loss of efficacy with extended dupilumab dosing intervals in many patients, indicating that reliable systemic UNLOAD and RESET strategies remain underdeveloped (60).

Psoriasis provides a second dermatological example. High-efficacy IL-17 and IL-23 blockade supports the CONTROL concept by demonstrating that deeper pathway suppression can achieve high rates of complete or near-complete clearance (29, 61). Selected de-escalation may be feasible in deep responders, as illustrated by the GUIDE trial, in which guselkumab interval extension was non-inferior in a predefined super-responder population (62). However, this does not imply that broad de-escalation is appropriate for all patients. Rather, psoriasis shows that UNLOAD and RESET may be possible only in selected patients with sustained deep response, while EQUILIBRATE is often approximated by continued scheduled maintenance therapy.

Bullous pemphigoid offers a further example in which the CURE logic is clinically intuitive but must be balanced against safety. High-potency topical corticosteroids or appropriately dosed systemic corticosteroids provide initial disease control, followed by structured tapering after cessation of new blister formation and transition to low-dose or steroid-sparing maintenance when needed (63–67). In this setting, the practical challenge is not only relapse prevention, but minimizing cumulative corticosteroid toxicity in an elderly and comorbidity-burdened population.

Chronic spontaneous urticaria may also be interpreted through the CURE lens, particularly because treatment often involves escalation to high-dose antihistamines or omalizumab, followed by gradual interval extension or discontinuation attempts in controlled patients (68–72). However, validated biomarkers defining safe RESET or EQUILIBRATE thresholds remain limited, making CSU a useful example of clinical phase-based practice without fully developed biological endpoints.

### 3.3 Conditions illustrating limits of reversibility

Hidradenitis suppurativa illustrates an important boundary condition for CURE. Unlike atopic dermatitis or psoriasis, HS is often diagnosed after years of active disease, when sinus tracts, fibrosis, scarring, and altered tissue architecture have already developed (73–75). At this stage, inflammatory suppression may reduce disease activity but cannot reliably restore normal tissue structure. TNF, IL-17, and dual IL-17A/F inhibition improve clinical outcomes in moderate-to-severe HS, but complete remission remains uncommon and treatment interruption is frequently followed by recurrence (76–80). Within the CURE framework, HS therefore illustrates that CONTROL may be only partially achievable, UNLOAD and RESET are rarely established, and EQUILIBRATE often requires continuous therapy. This does not contradict the model; rather, it emphasizes that phase-based reversibility depends strongly on disease stage, structural damage, and the timing of intervention.

### 3.4 Summary of clinical applicability

Across these examples, the applicability of CURE varies substantially. It is most clinically plausible where objective targets, biomarkers, pharmacokinetic monitoring, and de-escalation data already exist, as in Crohn’s disease and rheumatoid arthritis. It is partially reflected in dermatology, especially in topical atopic dermatitis, psoriasis, bullous pemphigoid, and chronic spontaneous urticaria, but systemic phase-tailored strategies remain incompletely developed. It is limited in diseases dominated by irreversible structural damage, such as advanced hidradenitis suppurativa. Additional threshold-oriented examples, such as asthma or hyperinflammatory syndromes, are summarized in **Table 3**. Accordingly, Chapter 3 is intended as an illustrative mapping of the CURE framework across selected clinical contexts, emphasizing patterns of alignment, current limitations, and areas requiring future validation.

## 4 Discussion

Across immune-mediated diseases, a consistent pattern emerges: durable disease control depends on decisive induction, guided de-escalation, and proactive maintenance. The relative importance and feasibility of each phase vary by disease, underlying pathophysiology, availability of biomarkers, and—critically—the timing of intervention.

The CURE framework does not replace disease-specific guidelines or treat-to-target strategies. Instead, it provides a unifying, phase-based logic grounded in systems immunology, explaining why early high-intensity therapy is often necessary, premature de-escalation leads to relapse, and maintenance above a critical threshold is required to stabilize immune networks. By integrating mechanistic insight with clinical practice across dermatologic, rheumatologic, gastroenterologic and hyperinflammatory conditions, CURE exposes both current best practices and their limitations, while defining priorities for future therapeutic development.

Ultimately, CURE reframes inflammation management from reactive symptom control toward threshold-aware, kinetic immune stabilization, offering a transferable conceptual language for personalized care and the long-term goal of functional remission with preserved tissue integrity.

### 4.1 What the CURE framework may help explain

Across diverse immune-mediated diseases, the CURE framework offers a unifying dynamic explanation for several recurring clinical observations: the frequent failure of symptom-driven therapy to achieve durable remission, the disproportionate benefit of early high-intensity intervention, and the high relapse rates observed after premature de-escalation or discontinuation of therapy.

Evidence from rheumatologic and gastrointestinal diseases demonstrates that crossing a disease-specific inflammatory threshold—rather than achieving incremental symptom improvement alone—is associated with deeper and more durable disease control. In Crohn’s disease, biomarker-guided escalation strategies in the CALM trial achieved significantly higher rates of endoscopic remission than symptom-based treatment adjustment, indicating that suppression of subclinical inflammatory activity is required to alter disease trajectory (47). Similarly, early combination therapy trials in rheumatoid arthritis (FIN-RACo, SWEFOT) demonstrated that rapid suppression of inflammation prevents irreversible joint damage, whereas delayed or step-up approaches allow destructive pathways to stabilize (50, 51, 81). These observations across immune-mediated diseases support the concept that therapeutic success depends on crossing a critical inflammatory threshold rather than merely reducing symptoms. Within the CURE framework, this principle explains why maintenance therapy remains necessary even after apparent clinical remission.

CURE may help explain why maintenance therapy remains necessary even after apparent remission. Across immune-mediated diseases, withdrawal studies consistently demonstrate substantial relapse rates despite clinical quiescence. For example, discontinuation of infliximab in Crohn’s disease (STORI) resulted in relapse in approximately 44% of patients within one year (48), while biologic withdrawal in rheumatoid arthritis similarly increased flare risk after remission had been achieved (7, 53–55, 82). These observations indicate that residual immune memory and feedback amplification can persist beyond symptom resolution, necessitating continued therapeutic pressure to maintain the system below the critical inflammatory threshold.

Importantly, CURE does not unify diseases at the level of molecular targets or pathways. Instead, it unifies them at the level of therapeutic dynamics—specifically, how timing, intensity, and duration of intervention interact with nonlinear immune networks to determine long-term outcomes.

### 4.2 Limitations of the CURE framework

Several limitations must be acknowledged. First, biomarkers that reliably define phase transitions are lacking in many diseases, particularly in atopic dermatitis and hidradenitis suppurativa. As a result, deep remission (TP2) cannot yet be universally operationalized: objective markers are needed to determine when de-escalation (dose reduction, interval extension, or discontinuation) can be pursued with acceptable relapse risk. Rather than assuming that symptom-based clearance equates to biological quiescence, CURE highlights a translational gap and frames TP2 as a prospective, indication-specific construct to be operationalized and validated using multimodal readouts (clinical measures, biomarkers, tissue/imaging, and PK/PD), with relapse hazard as the primary external validator (9, 15, 47).

Second, much of the supporting evidence is retrospective or *post hoc*, derived from trials not explicitly designed to test phase-based strategies. While de-escalation trials in Crohn’s disease and rheumatoid arthritis provide important proof-of-principle, prospective CURE-guided intervention studies are largely absent. Relatedly, CURE is not intended to replace disease-specific guidelines, treat-to-target algorithms, or molecularly defined therapeutic strategies. It does not prescribe specific drugs, dosing regimens, or biomarkers, nor does it imply that identical phase structures are achievable across all diseases.

Third, disease stage strongly modulates applicability, and the framework does not assume that all inflammatory diseases are equally reversible. Once irreversible structural damage dominates—as in advanced hidradenitis suppurativa or longstanding rheumatoid arthritis—crossing inflammatory thresholds may no longer translate into functional recovery, limiting the framework’s predictive power. Hyperinflammatory syndromes such as sepsis and cytokine release syndrome also differ fundamentally from chronic inflammatory diseases in timescale and reversibility, and are included only to illustrate extreme threshold behavior rather than chronic disease management (83–85).

Fourth, although the acronym CURE is intended as a concise mnemonic for CONTROL – UNLOAD and RESET – EQUILIBRATE, it should not be interpreted as implying definitive disease cure. For most chronic inflammatory diseases discussed here, the realistic therapeutic objective is not eradication of disease susceptibility, but stabilization of a state of low risk of relapse under the minimum effective therapeutic burden.

Finally, CURE does not yet account for interindividual variability in immune network topology, which may explain why identical therapeutic exposures yield divergent outcomes. Integrating genetic, epigenetic, and tissue-resident immune parameters remains an open challenge. Overall, CURE should not be interpreted as a normative algorithm: while the framework highlights associations between phase-appropriate therapy and outcomes, prospective validation of CURE-guided treatment strategies remains limited (9, 47, 86).

### 4.3 Conceptual implications: therapeutic kinetics over static targets

A central contribution of CURE is the shift from a static, target-centric view of inflammation control toward a kinetic, phase-aware perspective. Therapeutic success depends not only on which pathway is inhibited, but also on when, how intensely, and for how long suppression is applied relative to disease-specific thresholds.

Emerging CAR T-cell therapies in autoimmune diseases may represent an intensive example of therapeutic immune reset. Within CURE, CD19- or BCMA-directed CAR T-cell therapy could be interpreted as high-intensity CONTROL followed by UNLOAD and RESET through depletion of pathogenic B-cell or plasma-cell compartments and subsequent immune reconstitution. Persistent drug-free remission after reconstitution would conceptually resemble EQUILIBRATE. However, current evidence remains early, and CAR T-cell therapy should be viewed as a hypothesis-generating example rather than as validation of the CURE framework (87).

In this manuscript, therapeutic exposure is used as a conceptual term integrating treatment intensity, duration, timing, and pharmacodynamic depth. Operational correlates may include cumulative drug exposure, peak concentration, receptor occupancy, or time above a defined pharmacodynamic threshold. Such concepts are already applied in selected settings—for example, therapeutic drug monitoring strategies in Crohn’s disease that adjust infliximab dosing to predefined trough levels (88). However, comparable operational frameworks remain unavailable for most inflammatory diseases, highlighting a major translational gap.

Within this perspective, maintenance therapy represents not therapeutic inertia but an active stabilizing intervention required to maintain immune network equilibrium once control has been achieved (47, 54, 89–91).

### 4.4 Testable predictions and future directions

The CURE framework remains hypothesis-generating and requires prospective validation. All testable predictions and falsifiability criteria derived from the framework are consolidated in **Box 1**. These predictions translate the conceptual assumptions of CURE into operational hypotheses concerning threshold-like exposure–response relationships, early induction depth, deep remission before de-escalation, residual inflammatory activity, proactive maintenance, cross-indication phase dynamics, and phase-specific failure modes.

Future studies should test these predictions prospectively and in a disease-specific manner, using predefined clinical endpoints together with objective inflammatory readouts such as biomarkers, pharmacokinetic/pharmacodynamic parameters, imaging, tissue signatures, or validated disease activity measures. Evidence for nonlinear relationships between inflammatory suppression and relapse risk, lower relapse rates after de-escalation from objectively defined deep remission, and reduced cumulative disease burden under proactive maintenance would support the framework. Conversely, failure to demonstrate these relationships would weaken the CURE hypothesis and help define its boundaries.

### 4.5 Overall conclusion

In summary, the CURE framework integrates clinical experience and systems immunology to explain why durable inflammation control depends on decisive induction, guided de-escalation, and proactive maintenance. While the feasibility of each phase varies by disease, timing, and available biomarkers, the underlying dynamic principles recur across immune-mediated conditions.

CURE does not replace existing therapeutic paradigms but provides a conceptual scaffold that explains their successes and failures, highlights current limitations, and defines priorities for future research. By reframing inflammation management from reactive symptom suppression toward threshold-aware immune stabilization, CURE offers a transferable language for personalized care and the long-term goal of functional remission with preserved tissue integrity.

## Data Availability

The original contributions presented in the study are included in the article/supplementary material. Further inquiries can be directed to the corresponding author.

## References

[1] Serhan CN . Pro-resolving lipid mediators are leads for resolution physiology. Nature. (2014) 510:92–101. doi: 10.1038/nature13479 24899309 PMC4263681

[2] Schett G Neurath MF . Resolution of chronic inflammatory disease: universal and tissue-specific concepts. Nat Commun. (2018) 9:3261. doi: 10.1038/s41467-018-05800-6 30111884 PMC6093916

[3] Weidinger S Novak N . Atopic dermatitis. Lancet. (2016) 387:1109–22. doi: 10.1016/s0140-6736(15)00149-x 26377142

[4] Ghoreschi K Balato A Enerback C Sabat R . Therapeutics targeting the IL-23 and IL-17 pathway in psoriasis. Lancet. (2021) 397:754–66. doi: 10.1016/s0140-6736(21)00184-7 33515492

[5] McInnes IB Schett G . The pathogenesis of rheumatoid arthritis. N Engl J Med. (2011) 365:2205–19. doi: 10.1056/nejmra1004965 22150039

[6] Levy BD Clish CB Schmidt B Gronert K Serhan CN . Lipid mediator class switching during acute inflammation: signals in resolution. Nat Immunol. (2001) 2:612–9. doi: 10.1038/89759 11429545

[7] Schakel K Reich K Asadullah K Pinter A Jullien D Weisenseel P . Early disease intervention with guselkumab in psoriasis leads to a higher rate of stable complete skin clearance ('clinical super response'): Week 28 results from the ongoing phase IIIb randomized, double-blind, parallel-group, GUIDE study. J Eur Acad Dermatol Venereol. (2023) 37:2016–27. doi: 10.1111/jdv.19236 37262309

[8] Dignass A Ainsworth C Hartz S Dunnewind N Redondo I Sapin C . Efficacy and safety of advanced therapies in moderately-to-severely active ulcerative colitis: a systematic review and network meta-analysis. Adv Ther. (2024) 41:4446–62. doi: 10.1007/s12325-024-03003-8 39404996 PMC11550281

[9] Vande Casteele N Ferrante M Van Assche G Ballet V Compernolle G Van Steen K . Trough concentrations of infliximab guide dosing for patients with inflammatory bowel disease. Gastroenterology. (2015) 148:1320–9:e3. doi: 10.1053/j.gastro.2015.02.031 25724455

[10] Reddel HK Bacharier LB Bateman ED Brightling CE Brusselle GG Buhl R . Global Initiative for Asthma Strategy 2021: Executive summary and rationale for key changes. J Allergy Clin Immunol Pract. (2022) 10:S1–S18. doi: 10.1016/j.jaip.2021.10.001 34718211

[11] O'Byrne PM FitzGerald JM Bateman ED Barnes PJ Zhong N Keen C . Inhaled combined budesonide-formoterol as needed in mild asthma. N Engl J Med. (2018) 378:1865–76. doi: 10.1056/NEJMoa1715274 29768149

[12] Bateman ED Reddel HK O'Byrne PM Barnes PJ Zhong N Keen C . As-needed budesonide-formoterol versus maintenance budesonide in mild asthma. N Engl J Med. (2018) 378:1877–87. doi: 10.1056/nejmoa1715275 29768147

[13] Cheuk S Schlums H Gallais Serezal I Martini E Chiang SC Marquardt N . CD49a expression defines tissue-resident CD8(+) T cells poised for cytotoxic function in human skin. Immunity. (2017) 46:287–300. doi: 10.21417/b76k56 28214226 PMC5337619

[14] Cheuk S Wiken M Blomqvist L Nylen S Talme T Stahle M . Epidermal Th22 and Tc17 cells form a localized disease memory in clinically healed psoriasis. J Immunol. (2014) 192:3111–20. doi: 10.4049/jimmunol.1302313 24610014 PMC3962894

[15] Turner D Ricciuto A Lewis A D'Amico F Dhaliwal J Griffiths AM . STRIDE-II: An update on the Selecting Therapeutic Targets in Inflammatory Bowel Disease (STRIDE) Initiative of the International Organization for the Study of IBD (IOIBD): Determining therapeutic goals for treat-to-target strategies in IBD. Gastroenterology. (2021) 160:1570–83. doi: 10.1053/j.gastro.2020.12.031 33359090

[16] Wollenberg A Ehmann LM . Long term treatment concepts and proactive therapy for atopic eczema. Ann Dermatol. (2012) 24:253–60. doi: 10.5021/ad.2012.24.3.253 22879707 PMC3412232

[17] Kennedy NA Heap GA Green HD Hamilton B Bewshea C Walker GJ . Predictors of anti-TNF treatment failure in anti-TNF-naive patients with active luminal Crohn's disease: a prospective, multicentre, cohort study. Lancet Gastroenterol Hepatol. (2019) 4:341–53. doi: 10.1016/s2468-1253(19)30012-3 30824404

[18] Scheffer M Carpenter S Foley JA Folke C Walker B . Catastrophic shifts in ecosystems. Nature. (2001) 413:591–6. doi: 10.1038/35098000 11595939

[19] Reynolds A Rubin J Clermont G Day J Vodovotz Y Bard Ermentrout G . A reduced mathematical model of the acute inflammatory response: I. Derivation of model and analysis of anti-inflammation. J Theor Biol. (2006) 242:220–36. doi: 10.1016/j.jtbi.2006.02.016 16584750

[20] Scheffer M Carpenter SR Lenton TM Bascompte J Brock W Dakos V . Anticipating critical transitions. Science. (2012) 338:344–8. doi: 10.1126/science.1225244 23087241

[21] Ferl GZ Theil FP Wong H . Physiologically based pharmacokinetic models of small molecules and therapeutic antibodies: a mini-review on fundamental concepts and applications. Biopharm Drug Dispos. (2016) 37:75–92. doi: 10.1002/bdd.1994 26461173

[22] Dinarello CA . Overview of the IL-1 family in innate inflammation and acquired immunity. Immunol Rev. (2018) 281:8–27. doi: 10.1111/imr.12621 29247995 PMC5756628

[23] Garlanda C Dinarello CA Mantovani A . The interleukin-1 family: back to the future. Immunity. (2013) 39:1003–18. doi: 10.1016/j.immuni.2013.11.010 24332029 PMC3933951

[24] Sieminska I Pieniawska M Grzywa TM . The immunology of psoriasis-current concepts in pathogenesis. Clin Rev Allergy Immunol. (2024) 66:164–91. doi: 10.1007/s12016-024-08991-7 38642273 PMC11193704

[25] Chiricozzi A Guttman-Yassky E Suarez-Farinas M Nograles KE Tian S Cardinale I . Integrative responses to IL-17 and TNF-alpha in human keratinocytes account for key inflammatory pathogenic circuits in psoriasis. J Invest Dermatol. (2011) 131:677–87. doi: 10.1007/978-3-662-45139-7_81 21085185

[26] Lande R Gregorio J Facchinetti V Chatterjee B Wang YH Homey B . Plasmacytoid dendritic cells sense self-DNA coupled with antimicrobial peptide. Nature. (2007) 449:564–9. doi: 10.1038/nature06116 17873860

[27] Johnston A Xing X Wolterink L Barnes DH Yin Z Reingold L . IL-1 and IL-36 are dominant cytokines in generalized pustular psoriasis. J Allergy Clin Immunol. (2017) 140:109–20. doi: 10.1016/j.jaci.2016.08.056 28043870 PMC5494022

[28] Towne JE Renshaw BR Douangpanya J Lipsky BP Shen M Gabel CA . Interleukin-36 (IL-36) ligands require processing for full agonist (IL-36alpha, IL-36beta, and IL-36gamma) or antagonist (IL-36Ra) activity. J Biol Chem. (2011) 286:42594–602. doi: 10.1074/jbc.m111.267922 21965679 PMC3234937

[29] Reich K Warren RB Lebwohl M Gooderham M Strober B Langley RG . Bimekizumab versus secukinumab in plaque psoriasis. N Engl J Med. (2021) 385:142–52. doi: 10.1056/nejmoa2102383 33891380

[30] Eichenfield LF Tom WL Berger TG Krol A Paller AS Schwarzenberger K . Guidelines of care for the management of atopic dermatitis: section 2. Management and treatment of atopic dermatitis with topical therapies. J Am Acad Dermatol. (2014) 71:116–32. doi: 10.1016/j.jaad.2026.03.053 24813302 PMC4326095

[31] Thaci D . Long-term data in the treatment of psoriasis. Br J Dermatol. (2008) 159:18–24. doi: 10.1111/j.1365-2133.2008.08781.x 18700911

[32] Blauvelt A de Bruin-Weller M Gooderham M Cather JC Weisman J Pariser D . Long-term management of moderate-to-severe atopic dermatitis with dupilumab and concomitant topical corticosteroids (LIBERTY AD CHRONOS): a 1-year, randomised, double-blinded, placebo-controlled, phase 3 trial. Lancet. (2017) 389:2287–303. doi: 10.1016/s0140-6736(17)31191-1 28478972

[33] Wollenberg A Barbarot S Bieber T Christen-Zaech S Deleuran M Fink-Wagner A . Consensus-based European guidelines for treatment of atopic eczema (atopic dermatitis) in adults and children: part II. J Eur Acad Dermatol Venereol. (2018) 32:850–78. doi: 10.1111/j.1468-3083.2012.04636.x 29878606

[34] Strober B Tada Y Mrowietz U Lebwohl M Foley P Langley RG . Bimekizumab maintenance of response through 3 years in patients with moderate-to-severe plaque psoriasis: results from the BE BRIGHT open-label extension trial. Br J Dermatol. (2023) 188:749–59. doi: 10.1093/bjd/ljad035 36967713

[35] Vong L Ferraz JG Dufton N Panaccione R Beck PL Sherman PM . Up-regulation of Annexin-A1 and lipoxin A(4) in individuals with ulcerative colitis may promote mucosal homeostasis. PloS One. (2012) 7:e39244. doi: 10.1371/journal.pone.0039244 22723974 PMC3377644

[36] Ortega A Duran P Garrido B Manzano A Navarro C Silva A . Specialized pro-resolving lipid mediators in pulmonary diseases: molecular and therapeutic implications. Molecules. (2025) 30. doi: 10.3390/molecules30102212 40430385 PMC12114278

[37] Smolen JS Landewe RBM Bijlsma JWJ Burmester GR Dougados M Kerschbaumer A . EULAR recommendations for the management of rheumatoid arthritis with synthetic and biological disease-modifying antirheumatic drugs: 2019 update. Ann Rheum Dis. (2020) 79:685–99. doi: 10.1136/annrheumdis-2019-216655 31969328

[38] Hirten RP Lakatos PL Halfvarson J Colombel JF . A user's guide to de-escalating immunomodulator and biologic therapy in inflammatory bowel disease. Clin Gastroenterol Hepatol. (2020) 18:1336–45. doi: 10.1016/j.cgh.2019.12.019 31887444

[39] Gerss J Tedy M Klein A Horneff G Miranda-Garcia M Kessel C . Prevention of disease flares by risk-adapted stratification of therapy withdrawal in juvenile idiopathic arthritis: results from the PREVENT-JIA trial. Ann Rheum Dis. (2022) 81:990–7. doi: 10.1136/annrheumdis-2021-222029 35260388 PMC9209679

[40] Ravelli A Consolaro A Horneff G Laxer RM Lovell DJ Wulffraat NM . Treating juvenile idiopathic arthritis to target: recommendations of an international task force. Ann Rheum Dis. (2018) 77:819–28. doi: 10.1136/annrheumdis-2018-213030 29643108

[41] Barreto de Albuquerque J Mueller C Gungor B . Tissue-resident T cells in chronic relapsing-remitting intestinal disorders. Cells. (2021) 10. doi: 10.3390/cells10081882 34440651 PMC8393248

[42] Samat AAK van der Geest J Vastert SJ van Loosdregt J van Wijk F . Tissue-resident memory T cells in chronic inflammation-local cells with systemic effects? Cells. (2021) 10. doi: 10.3390/cells10020409 33669367 PMC7920248

[43] Bonasia CG Abdulahad WH Rutgers A Heeringa P Bos NA . B cell activation and escape of tolerance checkpoints: recent insights from studying autoreactive B cells. Cells. (2021) 10. doi: 10.3390/cells10051190 34068035 PMC8152463

[44] Shi JT Chen N Xu J Goyal H Wu ZQ Zhang JX . Diagnostic accuracy of fecal calprotectin for predicting relapse in inflammatory bowel disease: a meta-analysis. J Clin Med. (2023) 12. doi: 10.3390/jcm12031206 36769850 PMC9917450

[45] Murugesan N Saxena D Dileep A Adrish M Hanania NA . Update on the role of FeNO in asthma management. Diagnostics (Basel). (2023) 13. doi: 10.3390/diagnostics13081428 37189529 PMC10137365

[46] He H Olesen CM Pavel AB Clausen ML Wu J Estrada Y . Tape-strip proteomic profiling of atopic dermatitis on dupilumab identifies minimally invasive biomarkers. Front Immunol. (2020) 11:1768. doi: 10.3389/fimmu.2020.01768 32849633 PMC7423990

[47] Colombel JF Panaccione R Bossuyt P Lukas M Baert F Vanasek T . Effect of tight control management on Crohn's disease (CALM): a multicentre, randomised, controlled phase 3 trial. Lancet. (2017) 390:2779–89. doi: 10.1016/s0140-6736(17)32641-7 29096949

[48] Louis E Mary JY Vernier-Massouille G Grimaud JC Bouhnik Y Laharie D . Maintenance of remission among patients with Crohn's disease on antimetabolite therapy after infliximab therapy is stopped. Gastroenterology. (2012) 142:63–70:e5. doi: 10.1053/j.gastro.2011.09.034 21945953

[49] Louis E Resche-Rigon M Laharie D Satsangi J Ding N Siegmund B . Withdrawal of infliximab or concomitant immunosuppressant therapy in patients with Crohn's disease on combination therapy (SPARE): a multicentre, open-label, randomised controlled trial. Lancet Gastroenterol Hepatol. (2023) 8:215–27. doi: 10.1016/s2468-1253(22)00385-5 36640794 PMC9908559

[50] Mottonen T Hannonen P Leirisalo-Repo M Nissila M Kautiainen H Korpela M . Comparison of combination therapy with single-drug therapy in early rheumatoid arthritis: a randomised trial. FIN-RACo trial group. Lancet. (1999) 353:1568–73. doi: 10.1016/s0140-6736(98)08513-4 10334255

[51] van Vollenhoven RF Geborek P Forslind K Albertsson K Ernestam S Petersson IF . Conventional combination treatment versus biological treatment in methotrexate-refractory early rheumatoid arthritis: 2 year follow-up of the randomised, non-blinded, parallel-group Swefot trial. Lancet. (2012) 379:1712–20. doi: 10.1016/s0140-6736(12)60027-0 22464340

[52] Tanaka Y Hirata S Kubo S Fukuyo S Hanami K Sawamukai N . Discontinuation of adalimumab after achieving remission in patients with established rheumatoid arthritis: 1-year outcome of the HONOR study. Ann Rheum Dis. (2015) 74:389–95. doi: 10.1136/annrheumdis-2013-204016 24288014 PMC4316845

[53] Smolen JS Nash P Durez P Hall S Ilivanova E Irazoque-Palazuelos F . Maintenance, reduction, or withdrawal of etanercept after treatment with etanercept and methotrexate in patients with moderate rheumatoid arthritis (PRESERVE): a randomised controlled trial. Lancet. (2013) 381:918–29. doi: 10.1016/s0140-6736(12)61811-x 23332236

[54] Huizinga TW Conaghan PG Martin-Mola E Schett G Amital H Xavier RM . Clinical and radiographic outcomes at 2 years and the effect of tocilizumab discontinuation following sustained remission in the second and third year of the ACT-RAY study. Ann Rheum Dis. (2015) 74:35–43. doi: 10.1136/annrheumdis-2014-205752 25169728 PMC4283697

[55] Tanaka Y Smolen JS Jones H Szumski A Marshall L Emery P . The effect of deep or sustained remission on maintenance of remission after dose reduction or withdrawal of etanercept in patients with rheumatoid arthritis. Arthritis Res Ther. (2019) 21:164. doi: 10.1186/s13075-019-1937-4 31277720 PMC6610967

[56] Wollenberg A Reitamo S Atzori F Lahfa M Ruzicka T Healy E . Proactive treatment of atopic dermatitis in adults with 0.1% tacrolimus ointment. Allergy. (2008) 63:742–50. doi: 10.1111/j.1398-9995.2008.01683.x 18445188

[57] Breneman D Fleischer AB Abramovits W Zeichner J Gold MH Kirsner RS . Intermittent therapy for flare prevention and long-term disease control in stabilized atopic dermatitis: a randomized comparison of 3-times-weekly applications of tacrolimus ointment versus vehicle. J Am Acad Dermatol. (2008) 58:990–9. doi: 10.1016/j.jaad.2008.02.008 18359127

[58] Hanifin J Gupta AK Rajagopalan R . Intermittent dosing of fluticasone propionate cream for reducing the risk of relapse in atopic dermatitis patients. Br J Dermatol. (2002) 147:528–37. doi: 10.1046/j.1365-2133.2002.05006.x 12207596

[59] Liu L Ong G . A randomized, open-label study to evaluate an intermittent dosing regimen of fluticasone propionate 0.05% cream in combination with regular emollient skin care in reducing the risk of relapse in pediatric patients with stabilized atopic dermatitis. J Dermatolog Treat. (2018) 29:501–9. doi: 10.1080/09546634.2017.1401211 29164960

[60] Worm M Simpson EL Thaci D Bissonnette R Lacour JP Beissert S . Efficacy and safety of multiple dupilumab dose regimens after initial successful treatment in patients with atopic dermatitis: a randomized clinical trial. JAMA Dermatol. (2020) 156:131–43. doi: 10.1001/jamadermatol.2019.3617 31876900 PMC6990756

[61] Lebwohl MG Merola JF Rowland K Miller M Yang YW Yu J . Safety of guselkumab treatment for up to 5 years in patients with moderate-to-severe psoriasis: pooled analyses across seven clinical trials with more than 8600 patient-years of exposure. Br J Dermatol. (2023) 189:42–52. doi: 10.1093/bjd/ljad115 37022762

[62] Eyerich K Asadullah K Pinter A Weisenseel P Reich K Paul C . Noninferiority of 16-week vs 8-week guselkumab dosing in super responders for maintaining control of psoriasis: the GUIDE randomized clinical trial. JAMA Dermatol. (2024) 160:953–63. doi: 10.1001/jamadermatol.2024.2463 39083288 PMC11292573

[63] Joly P Roujeau JC Benichou J Picard C Dreno B Delaporte E . A comparison of oral and topical corticosteroids in patients with bullous pemphigoid. N Engl J Med. (2002) 346:321–7. doi: 10.1056/nejmoa011592 11821508

[64] Borradori L Van Beek N Feliciani C Tedbirt B Antiga E Bergman R . Updated S2 K guidelines for the management of bullous pemphigoid initiated by the European Academy of Dermatology and Venereology (EADV). J Eur Acad Dermatol Venereol. (2022) 36:1689–704. doi: 10.1007/978-3-662-45698-9_55 35766904

[65] Murrell DF Daniel BS Joly P Borradori L Amagai M Hashimoto T . Definitions and outcome measures for bullous pemphigoid: recommendations by an international panel of experts. J Am Acad Dermatol. (2012) 66:479–85. doi: 10.1016/j.jaad.2011.06.032 22056920 PMC3883429

[66] Hebert V Bastos S Drenovska K Meijer J Ingen-Housz-Oro S Bedane C . International multicentre observational study to assess the efficacy and safety of a 0.5 mg kg(-1) per day starting dose of oral corticosteroids to treat bullous pemphigoid. Br J Dermatol. (2021) 185:1232–9. doi: 10.1111/bjd.20593 34173243

[67] Polansky M Eisenstadt R DeGrazia T Zhao X Liu Y Feldman R . Rituximab therapy in patients with bullous pemphigoid: a retrospective study of 20 patients. J Am Acad Dermatol. (2019) 81:179–86. doi: 10.1016/j.jaad.2019.03.049 30923002

[68] Zuberbier T Abdul Latiff AH Abuzakouk M Aquilina S Asero R Baker D . The international EAACI/GA(2)LEN/EuroGuiDerm/APAAACI guideline for the definition, classification, diagnosis, and management of urticaria. Allergy. (2022) 77:734–66. doi: 10.1111/all.15090 34536239

[69] Sanchez J Alvarez L Cardona R . Prospective analysis of clinical evolution in chronic urticaria: persistence, remission, recurrence, and pruritus alone. World Allergy Organ J. (2022) 15:100705. doi: 10.1016/j.waojou.2022.100705 36267098 PMC9554810

[70] Maurer M Rosen K Hsieh HJ Saini S Grattan C Gimenez-Arnau A . Omalizumab for the treatment of chronic idiopathic or spontaneous urticaria. N Engl J Med. (2013) 368:924–35. doi: 10.1056/nejmoa1215372 23432142

[71] Asero R Calzari P Vaienti S Cugno M . Therapies for chronic spontaneous urticaria: present and future developments. Pharm (Basel). (2024) 17. doi: 10.3390/ph17111499 39598410 PMC11597230

[72] Metz M Staubach P Bauer A Brehler R Gericke J Kangas M . Clinical efficacy of omalizumab in chronic spontaneous urticaria is associated with a reduction of FcepsilonRI-positive cells in the skin. Theranostics. (2017) 7:1266–76. doi: 10.1097/aci.0b013e328355365a 28435464 PMC5399592

[73] Zouboulis CC Desai N Emtestam L Hunger RE Ioannides D Juhasz I . European S1 guideline for the treatment of hidradenitis suppurativa/acne inversa. J Eur Acad Dermatol Venereol. (2015) 29:619–44. doi: 10.1111/jdv.12966 25640693

[74] Saunte DML Jemec GBE . Hidradenitis suppurativa: advances in diagnosis and treatment. JAMA. (2017) 318:2019–32. doi: 10.1001/jama.2017.16691 29183082

[75] Jemec GB . Clinical practice. Hidradenitis suppurativa. N Engl J Med. (2012) 366:158–65. doi: 10.1007/s10227-002-2104-z 22236226

[76] Kimball AB Okun MM Williams DA Gottlieb AB Papp KA Zouboulis CC . Two phase 3 trials of adalimumab for hidradenitis suppurativa. N Engl J Med. (2016) 375:422–34. doi: 10.1056/nejmoa1504370 27518661

[77] Kimball AB Bechara FG Badat A Giamarellos-Bourboulis EJ Gottlieb AB Jemec GBE . Long-term efficacy and safety of secukinumab in patients with moderate-to-severe hidradenitis suppurativa: week 104 results from the SUNSHINE and SUNRISE extension trial. Br J Dermatol. (2025) 192:629–40. doi: 10.1093/bjd/ljae469 39611771

[78] Kimball AB Jemec GBE Sayed CJ Kirby JS Prens E Ingram JR . Efficacy and safety of bimekizumab in patients with moderate-to-severe hidradenitis suppurativa (BE HEARD I and BE HEARD II): two 48-week, randomised, double-blind, placebo-controlled, multicentre phase 3 trials. Lancet. (2024) 403:2504–19. doi: 10.1016/s0140-6736(24)00101-6 38795716

[79] Abdalla T Lowes MA Kaur N Micheletti RG Steinhart AH Alavi A . Is there a role for therapeutic drug monitoring in patients with hidradenitis suppurativa on tumor necrosis factor-alpha inhibitors? Am J Clin Dermatol. (2021) 22:139–47. doi: 10.1007/s40257-020-00579-z 33398848

[80] Zouboulis CC Okun MM Prens EP Gniadecki R Foley PA Lynde C . Long-term adalimumab efficacy in patients with moderate-to-severe hidradenitis suppurativa/acne inversa: 3-year results of a phase 3 open-label extension study. J Am Acad Dermatol. (2019) 80:60–9. doi: 10.1016/j.jaad.2018.05.040 29860040

[81] Bergstra SA Landewe RBM Huizinga TWJ Allaart CF . Rheumatoid arthritis patients with continued low disease activity have similar outcomes over 10 years, regardless of initial therapy. Rheumatol (Oxford). (2017) 56:1721–8. doi: 10.1136/annrheumdis-2017-eular.1707 28957556

[82] Smolen JS Landewe R Bijlsma J Burmester G Chatzidionysiou K Dougados M . EULAR recommendations for the management of rheumatoid arthritis with synthetic and biological disease-modifying antirheumatic drugs: 2016 update. Ann Rheum Dis. (2017) 76:960–77. doi: 10.1136/annrheumdis-2016-210715 28264816

[83] Kang S Tanaka T Narazaki M Kishimoto T . Targeting interleukin-6 signaling in clinic. Immunity. (2019) 50:1007–23. doi: 10.1016/j.immuni.2019.03.026 30995492

[84] Nishimoto N Kanakura Y Aozasa K Johkoh T Nakamura M Nakano S . Humanized anti-interleukin-6 receptor antibody treatment of multicentric Castleman disease. Blood. (2005) 106:2627–34. doi: 10.1182/blood-2004-12-4602 15998837

[85] Hotchkiss RS Moldawer LL Opal SM Reinhart K Turnbull IR Vincent JL . Sepsis and septic shock. Nat Rev Dis Primers. (2016) 2:16045. doi: 10.1038/nrdp.2016.45 28117397 PMC5538252

[86] de Jong MJ Huibregtse R Masclee AAM Jonkers D Pierik MJ . Patient-reported outcome measures for use in clinical trials and clinical practice in inflammatory bowel diseases: a systematic review. Clin Gastroenterol Hepatol. (2018) 16:648–53. doi: 10.1016/j.cgh.2017.10.019 29074448

[87] Muller F Taubmann J Bucci L Wilhelm A Bergmann C Volkl S . CD19 CAR T-cell therapy in autoimmune disease - a case series with follow-up. N Engl J Med. (2024) 390:687–700. doi: 10.1056/NEJMoa2308917 38381673

[88] Pouillon L Ferrante M Van Assche G Rutgeerts P Noman M Sabino J . Mucosal healing and long-term outcomes of patients with inflammatory bowel diseases receiving clinic-based vs trough concentration-based dosing of infliximab. Clin Gastroenterol Hepatol. (2018) 16:1276–83. doi: 10.1016/j.cgh.2017.11.046 29203225

[89] Smolen JS Breedveld FC Burmester GR Bykerk V Dougados M Emery P . Treating rheumatoid arthritis to target: 2014 update of the recommendations of an international task force. Ann Rheum Dis. (2016) 75:3–15. doi: 10.1136/annrheumdis-2015-207524 25969430 PMC4717393

[90] Burgers LE Raza K van der Helm-van Mil AH . Window of opportunity in rheumatoid arthritis - definitions and supporting evidence: from old to new perspectives. RMD Open. (2019) 5:e000870. doi: 10.1136/rmdopen-2018-000870 31168406 PMC6525606

[91] Girolomoni G Griffiths CE Krueger J Nestle FO Nicolas JF Prinz JC . Early intervention in psoriasis and immune-mediated inflammatory diseases: a hypothesis paper. J Dermatolog Treat. (2015) 26:103–12. doi: 10.3109/09546634.2014.880396 24547907

[92] Makowska K Nowaczyk J Samochocki Z Blicharz L Rudnicka L . Topical proactive therapy in dermatology. A scoping review. Postepy Dermatol Alergol. (2023) 40:510–7. doi: 10.5114/ada.2023.129454 37692271 PMC10485751

[93] Wollenberg A Bieber T . Proactive therapy of atopic dermatitis--an emerging concept. Allergy. (2009) 64:276–8. doi: 10.1111/j.1398-9995.2008.01803.x 19076538

[94] Zouboulis CC Bechara FG Benhadou F Bettoli V Bukvic Mokos Z Del Marmol V . European S2k guidelines for hidradenitis suppurativa/acne inversa part 2: treatment. J Eur Acad Dermatol Venereol. (2025) 39:899–941. doi: 10.1111/jdv.20472 39699926 PMC12023723

[95] Miotto ESVB Mitraud SAV Furtado RNV Natour J Len CA Terreri M . Patients with juvenile idiopathic arthritis in clinical remission with positive power Doppler signal in joint ultrasonography have an increased rate of clinical flare: a prospective study. Pediatr Rheumatol Online J. (2017) 15:80. doi: 10.1186/s12969-017-0208-7 29132381 PMC5683235

[96] Rabe KF Nair P Brusselle G Maspero JF Castro M Sher L . Efficacy and safety of dupilumab in glucocorticoid-dependent severe asthma. N Engl J Med. (2018) 378:2475–85. doi: 10.1056/nejmoa1804093 29782224

